# The molecular basis of talin2’s high affinity toward β1-integrin

**DOI:** 10.1038/srep41989

**Published:** 2017-02-03

**Authors:** Yaxia Yuan, Liqing Li, Yanyan Zhu, Lei Qi, Latifeh Azizi, Vesa P. Hytönen, Chang-Guo Zhan, Cai Huang

**Affiliations:** 1 Molecular Modeling and Biopharmaceutical Center, College of Pharmacy, University of Kentucky, Lexington, KY 40506, USA; 2 Markey Cancer Center, University of Kentucky, Lexington, KY 40506, USA; 3 BioMediTech, University of Tampere, 33520 Tampere, Finland and Fimlab Laboratories, 33520 Tampere, Finland; 4 Department of Pharmacology and nutritional sciences, University of Kentucky, Lexington, KY 40506, USA

## Abstract

Talin interacts with β-integrin tails and actin to control integrin activation, thus regulating focal adhesion dynamics and cell migration. There are two talin genes, *Tln1* and *Tln2*, which encode talin1 and talin2, and it is generally believed that talin2 functions redundantly with talin1. However, we show here that talin2 has a higher affinity to β1-integrin tails than talin1. Mutation of talin2 S339 to leucine, which can cause Fifth Finger Camptodactyly, a human genetic disease, completely disrupted its binding to β–integrin tails. Also, substitution of talin1 C336 with Ser enhanced the affinity of talin1, whereas substitution of talin2 S339 with Cys diminished that of talin2. Further computational modeling analysis shows that talin2 S339 formed a hydrogen bond with E353, which is critical for inducing key hydrogen bonds between talin2 N326 and β1-integrin R760, and between talin2 K327 and β1-integrin D759. Mutation at any of these residues significantly diminished the interaction of talin2 with β1- integrin tails. These hydrogen bonds were not observed in talin1/β1-integrin, but did exist in talin1^C336S^/β1-integrin complex. These results suggest that talin2 S339 forms a hydrogen bond with E353 to mediate its high affinity to β1-integrin.

Integrins are a family of transmembrane adhesion receptors that mediate cell-matrix and cell-cell adhesion^1^. Integrins are heterodimers, comprising α (alpha) and β (beta) subunits. Talin, a large focal adhesion protein, binds to the beta subunit, consequently activating integrin, which in turn regulates cell migration^2^,^3^, invasion^4^,^5^, growth^6^,^7^, differentiation^6^,^8^, and apoptosis^9^. As a result, integrin activation modulates a variety of physiological and pathological processes, such as development, immunity, inflammation, and tumor metastasis. Thus, the talin-integrin interaction is one of the most important protein-protein interactions.

Talin contains an amino-terminal globular head domain and a carboxy-terminal rod domain^10^. The talin head domain contains a FERM (band four-point-one, ezrin, radixin, moesin homology) domain, which comprises three subdomains, F1, F2 and F3. The F2 domain is entirely α-helical with a short linked region, and the F3 domain is a sandwich of two orthogonal antiparallel β-sheets followed by an α-helix^11^. The FERM domain is responsible for the binding of talin to β-integrin tails^12^, type I phosphatidylinositol 4-phosphate 5-kinase γ (PIPKIγ)^13^,^14^, and focal adhesion kinase (FAK)^15^. The rod domain has several vinculin-binding sites, and two actin-binding sites^16^.

The binding of talin to β integrin tails is essential for integrin activation^12^,^17^, which in turn regulates focal adhesion (FA) dynamics, a key step in cell migration^18^,^19^. Talin also mediates calpain-induced FA disassembly^19^. We and our collaborators have shown that talin phosphorylation by Cdk5 regulates FA dynamics, cell migration and invasion^20^,^21^. Talin interacts with PIPKIγ, which produces PIP_2_ to regulate FA dynamics, cell migration and invasion^13^,^14^,^22^,^23^,^24^,^25^,^26^,^27^,^28^.

There are two talin genes, *Tln1* and *Tln2*, encoding talin1 and talin2, respectively. Talin1 has been well studied, while the biological function of talin2 is less clear. It was presumed that talin2 functions redundantly with talin1. However, recent evidence indicates that talin2 is functionally different from talin1. Previous study shows that talin2 regulates focal adhesion assembly and focal adhesion kinase (FAK) signaling in the absence of talin1^29^. Talin2 is usually localized at large FAs and fibrillar adhesions, whereas talin1 is usually found at smaller FAs in the peripheral region^30^,^31^. Trastuzumab, a HER2-targeting antibody drug for cancer therapy, inhibits cell migration and invasion, most likely through down-regulating talin2^32^. Recently, we reported that talin2 binds to β integrin tails more strongly than talin1, and a strong interaction of talin2 with β integrins is required for the generation of traction force, which in turn drives invadopodium formation and cell invasion^33^. Nevertheless, the underlying molecular basis for the difference between talin1 and talin2 remains to be elucidated.

In the present study, we demonstrated that talin2 had a higher affinity to β1-integrin tails than talin1, talin2 Ser339 is largely responsible for this affinity difference, and mutation at Ser339 reduced its binding to β1-integrin tails. Coincidently, a new study shows that Fifth Finger Camptodactyly, a human genetic disease, is caused by a Leucine mutation at talin2 Ser339^34^. We defined the molecular basis of talin2 binding to β1-integrin using computational modeling methods, performed experiments to verify the computational model, and examined the role of talin2 S339 in focal adhesion assembly.

## Results

### Talin2 has a higher affinity to β1-integrin tails than talin1

**Figure 1:**
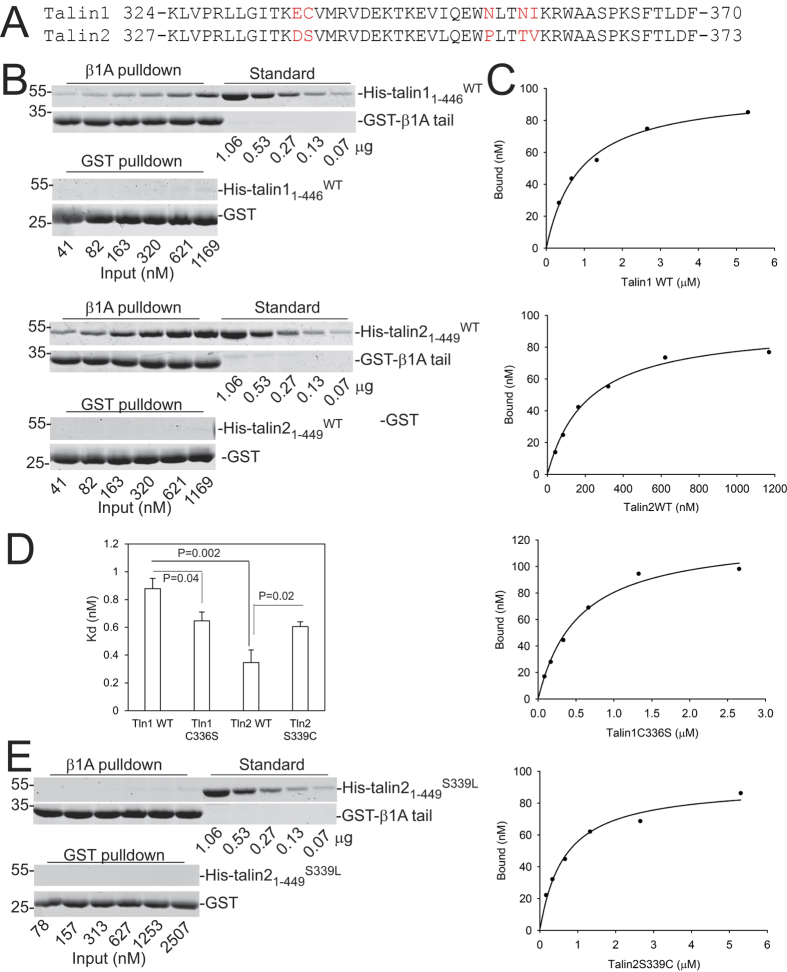
Affinities of talin1_1–446_, talin1_1–446_^C336S^, talin2_1–449_, and talin2_1–449_^S339C^ to β1-integrin tails. The binding of purified His-tagged proteins to GST-β1-integrin tails was determined using GST pulldown assays. Bound protein was separated using SDS-PAGE, stained with Coomassie blue, and calibrated with standards run on the same gels. The binding curves and the K_d_ were analyzed with SigmaPlot. (**A)** Sequence alignment of the integrin-binding region of the F3 domains of talin1 and talin2. Unmatched residues are shown in Red. (**B**) GST-β1 tails-bound talin1_1–446_ and talin2_1–449_ were compared after SDS-PAGE and Coomassie blue staining. Cropped gel images are shown. The full-length gel images are included in Supplementary Fig. S3. (**C**) The specific binding curves of talin1_1–446_, talin1_1–446_^C336S^, talin2_1–449_, and talin2_1–449_^S339C^ to β1-integrin tails. Please note that protein concentrations used in binding assays were different. (**D**) The dissociation constants of talin1_1–446_, talin1_1–446_^C336S^, talin2_1–449_, and talin2_1–449_^S339C^ binding to β1A-integrin tails. Data are presented as mean ± SEM. P values from student’s t-test are shown. (**E**) GST-β1 tails-bound talin2_1–449_^S339L^ were detected by Coomassie blue staining. Cropped gel images are shown. The full-length gel images are included in Supplementary Fig. S3.

Recently, we demonstrated that talin2 had a stronger binding to β-integrin tails than talin1^33^. There are several residues that are different between the integrin-binding sequences of talin1 and talin2 (Fig. 1A), but Cys336 of talin1 and the corresponding Ser339 on talin2 are largely responsible for their binding difference. Substitution of Cys336 with Ser enhanced talin1’s binding to β1A-integrin tails, whereas substitution of Ser339 with Cys or Leu significantly attenuated talin2 binding, suggesting the critical role of Ser339 in mediating talin2 binding to β-integrins.

To study the interaction of talin and mutants with β1-integrin tails, we purified His tagged talin1_1–446_^WT^, talin1_1–446_^C336S^, talin2_1–449_^WT^, and talin2_1–449_^S339C^ overexpressed in E. coli. To examine the quality of the proteins, purified proteins were analyzed by gel filtration chromatography combined with static light scattering detector. As shown in Supplementary Fig. S1 and Table S1, all proteins were mainly monomeric in solution (judged by static light scattering), with molecular weights approximately 59 kDa, which is consistent with theoretical molecular weights (~53 kDa). Also, the eluted protein peak was rather symmetric in the case of all the protein forms, suggesting that these proteins are soluble and folded.

To determine the affinity of talin1 to β1-integrin tails, different concentrations of His-tagged-talin1_1–446_ were incubated with glutathione agarose beads that were preloaded with GST-β1A-integrin tails. Unbound protein was washed away and bound protein was separated by SDS-PAGE, stained with Coomassie blue, and quantitated with standards run on the same gel. The β1A-integrin tails bound to talin1_1–446_ moderately, but GST did not (Fig. 1B). The dissociation constant (K_d_) between talin1_1–446_ and β1A-integrin tails is approx. 0.88 ± 0.07 μM (n = 5) (Fig. 1C,D). We employed the same method to determine the K_d_ between talin2_1–449_ and β1 A integrin tails. The K_d_ between talin2_1–449_ and β1A-integrin tails is approx. 0.35 ± 0.09 μM (n = 5) (Fig. 1B–1D). Substitution of talin1 C336 with Ser caused a slight increase in its affinity, whereas substitution of talin2 S339 with Cys diminished its affinity (Fig. 1C,D). Surprisingly, substitution of talin2 S339 with Leu completely abolished its interaction with β1 A integrin tails (Fig. 1E). These results indicate that S339 is critical for talin2’s high affinity for β integrins.

### Talin2 forms closer contacts and more hydrogen bonds with β1 integrin tails than talin1

**Figure 2:**
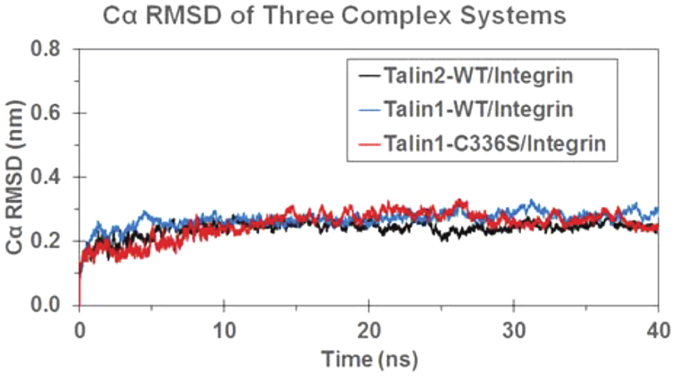
Tracked positional RMSD values for the wild type Talin1/Integrin, C336S mutant Talin1/Integrin and wild type Talin2/Integrin complex base on 40 ns MD simulation.

First, we compared the energy minimization and molecular dynamics (MD) in talin2^WT^/integrin, talin1^WT^/integrin, and talin1^C336S^/integrin complexes. Depicted in Fig. 2 are the structural dynamics tracked as positional root-mean square deviation (RMSD) of Cα atoms along the 40 ns MD simulations in the three talin/integrin complex structures. After a time period of ~10 ns, all the RMSD curves become flat, indicating the complex structure in the three MD systems are relaxed and equilibrated.

**Figure 3:**
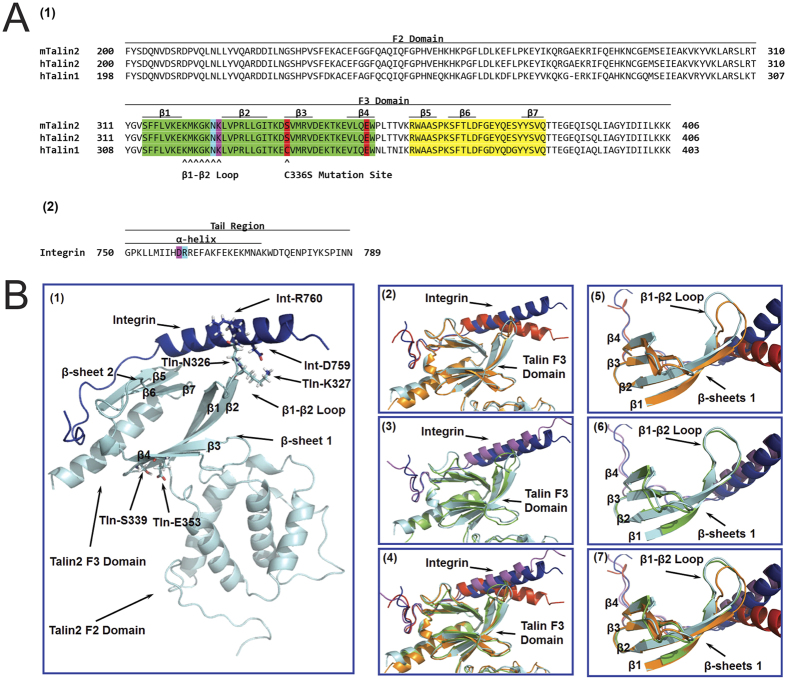
Superimposition of talin/integrin complexes. (**A**) Sequence alignment of *Mus musculus* talin2 (mTalin2), *Homo sapiens* talin2 (hTalin2), and *Homo sapiens* talin1 (hTalin1) by PROMALS3D server. β strands of talin (β1-β7), α helix of β1-integrin as well as the name of the subunit are marked by black lines. β-sheet 1 and β-sheet 2 are colored in green and yellow respectively. The key interaction pair of Tln-S339/336 and Tln-E353/350 are colored in red, the key interaction pair of Tln-K324/327 and Int-D759 are colored in purple, and the Tln-N323/326 and Int-R760 interaction pair are colored in cyan. (**B)** (1) Typical talin2^WT^/integrin complex structure is derived from the last snapshot of the 40 ns MD trajectory by energy minimization. Talin2 and β1-integrin are represented as cyan and blue ribbons, respectively. Tln-S339, Tln-E353, Tln-K327, Tln-N326, Int-R760, and Int-D759 are showed in stick-ball model. (2) Superimposition of the talin2^WT^/integrin and talin1^WT^/integrin complexes. For talin2^WT^/integrin complex, talin2^WT^ and β1-integrin are colored in cyan and blue, respectively. For talin1^WT^/integrin complex, talin1^WT^ and β1-integrin are colored in golden and red, respectively. (3) Only β-sheet 1 of talin and β1-intergrin are shown. Although other parts of talin could be superimposed well, β-sheet 1 varies a lot in talin2^WT^ and talin1^WT^. (4) Superimposition of the talin2^WT^/integrin and talin1^C336S^/integrin complex. Talin1^C336S^ and β1-integrin are colored in green and purple, respectively. (5) Only β-sheet 1 of talin and β1-integrin are shown, and they both have similar conformation in talin2^WT^/integrin and talin1^C336S^/integrin complexes. (6) Superimposition of all three talin/integrin complexes. (7) Only β-sheet 1 of talin and β1-intergin are shown.

The sequences of mouse talin2 (mTalin2), human talin2 (hTalin2), human talin1 (hTalin1) and human β1D-integrin tail are shown in Fig. 3A. To understand the structural basis of talin1 and talin2 binding to β1-integrin tail, we performed molecular modeling and MD simulations to explore the structural differences in talin1 and talin2. Briefly, the talin1/integrin tail complex structure is constructed based on talin2/integrin complex structure^11^ with an homology modeling method implemented by the MODELLER module of Discovery Studio 2.5 as described previously^35^,^36^. As shown in Fig. 3B1, the β1-integrin tail mostly contacts with these two β-sheets of F3 domain. Based on the energy minimized complex structures from 40 ns MD simulation, we found that the β1-integrin tail binds to talin1^WT^ with a very different orientation comparing with its binding to talin2^WT^ (Fig. 3B2), while the single mutation C336S of talin1 switches the β1-integrin in Talin1^WT^/integrin complex to a similar orientation seen in talin2^WT^/integrin complex (Fig. 3B3,B4). Furthermore, the average structures of the talin2^WT^/integrin, talin1^WT^/integrin, and talin1^C336S^/integrin complexes of 40 ns simulation also confirm the systematic difference between talin2^WT^/integrin and talin1^WT^/integrin and the systematic similarity between talin2^WT^/integrin and talin1^C336S^/integrin (Supplementary Fig. S2). Interestingly, the β-sheet 1, especially the loop region between β strand 1 (β1) and β strand 2 (β2) in β-sheet 1 adopts significantly different conformations in talin2^WT^/integrin and talin1^WT^/integrin complex (Fig. 3B5), while the C336S mutant of talin1 eliminates the difference (Fig. 3B6,3B**7**). This is consistent with experimental results that talin2 has a higher affinity to β1-integrin tails than talin1, and that the substitution of talin1 C336 with Ser enhances the affinity of talin1 (Fig. 1).

### Role of S339 in talin/integrin interaction

**Figure 4:**
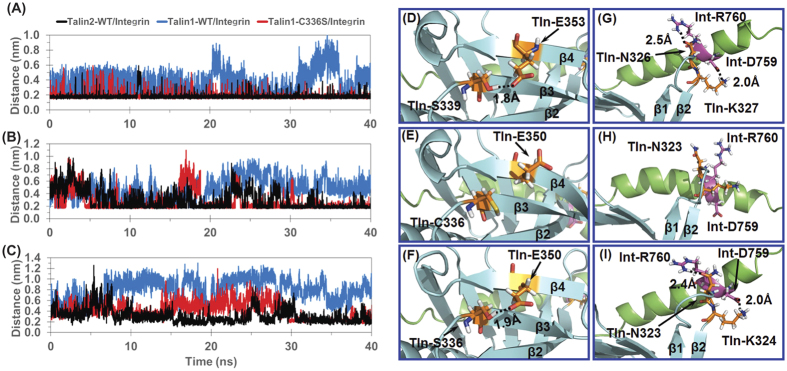
The hydrogen bonds between Tln-S339 and Tln-E353, Tln-K327 and Int-D759, and Tln-N326 and Int-R760 contribute to their affinity to β1-integrins. (**A**) Tracked distance of the intra-molecular hydrogen bond formed by residue S336/339 and E350/353 of talin. Black, blue, and red tracks represent the distance (Unit in Å) between the Hɣ atom of S339/C336/S336 and the Oε atom of E353/350 in talin2^WT^/integrin, talin1^WT^/integrin, and talin1^C336S^/integrin complexes, respectively. (**B**) Tracked distance of the inter-molecular hydrogen bond formed between the positive-charged side chain of K324/327 in talin and the negative charged side chain of D759 in β1-integrin. Black, blue, and red tracks represent the distance between the Hζ atom of K327/324 of talin and the Oδ atom at D759 of β1-integrin in talin2^WT^/integrin, talin1^WT^/integrin, talin1^C336S^/integrin complexes, respectively. (**C**) Tracked distance of the inter-molecular hydrogen bond formed between the side chain of N323/326 in talin and side chain of R760 in β1-integrin. Black, blue, and red tracks represent the distance between the Oδ atom of N326/323 of talin and the Hη atom of R760 of β1-integrin in talin2^WT^/integrin, talin1^WT^/integrin, and talin1^C336S^/integrin complexes, respectively. (**D**) Hydrogen bond between S339 and E353 of talin with labeled distance in talin2^WT^/integrin complex. Talin is represent as cyan ribbons. S339 and E353 are shown in golden stick-ball style. (**E**) Side chains of C336 and E350 are far away from each other in talin1^WT^/integrin complex. (**F**) Hydrogen bond between S336 and E350 with labeled distance in talin1^C336S^/integrin complex. (**G**) Hydrogen bond between Tln-K327 and Int-D759, and Tln-N326 and Int-R760 with labeled distance in talin2^WT^/integrin complex. Talin and β1-integrin are presented as cyan ribbons and green ribbons respectively. (**H**) Side chains of Tln-K324, Tln-N323, Int-D759, and Int-R760 are far away from each other in talin1^WT^/integrin complex. (**I**) Hydrogen bond between Tln-K324 and Int-D759, and Tln-N323 and Int-R760 with labeled distance in talin1^C336S^/integrin complex.

As the β1-β2 loop of talin contacts directly with β1-integrin, conformation of β-sheet 1 should be one of the decisive factors for the orientation of β1-integrin binding with talin. However, β-sheet 1 of talin1 and talin2 are fully conserved except in two residues, D338 and S339 in talin2^WT^ corresponding to E335 and C336 in talin1^WT^, respectively (Fig. 3A). As the side chains of aspartic acid and glutamic acid have similar chemical property, S339 of talin2^WT^ and C336 of talin1^WT^ could be considered as the major difference in β-sheet 1 of the two isoforms. As a result, we expect that the C336S mutation of talin1^WT^ would adapt the conformation of β-sheet 1 similar to that in talin2^WT^, thus talin1^C336S^ and talin2^WT^ would bind to β1-integrin with similar binding orientation. Fig. 4A depicts the tracked distances and typical snapshots of the hydrogen bond between S336/339 and E350/353 in talin/integrin complex from the MD trajectories (residue number with slash indicates residue in talin1/talin2, respectively). The average distance of this hydrogen bond (in the last 10 ns of simulation) is 1.75 ± 0.18 Å (frequency of hydrogen bond occurrence: 99.3%, with 2.5 Å H-O distance threshold) and 1.78 ± 0.24 Å (frequency: 98.2%) in talin1^C336S^/integrin and talin2^WT^/integrin complex respectively, while a much larger and more fluctuating average distance of 4.39 ± 1.92 Å (frequency: 27.0%) between terminus hydrogen of C336 and oxygen of carboxyl group of E350 is observed in talin1^WT^/integrin complex. Thus, the hydrogen bond between S339 and E353 in talin2^WT^ does not exist in talin1^WT^ due to the weak hydrogen bond capacity of corresponding residue C336 in talin1^WT^ comparing with S339 in talin2^WT^ (Fig. 4D,E). Therefore, C336S mutation of Talin1 is expected to restore this hydrogen bond (Fig. 4F). In addition, as residues S336/339 and E350/353 could form hydrogen bond between the β3 and β4 strand of β-sheet 1, loss of this strong interaction in talin1^WT^/integrin complex would influence the steric alignment of β strands in β-sheet 1, which is also observed in the typical snapshots from MD simulation (Fig. 3B5).

### The hydrogen bonds between talin and β1-integrin contribute to talin’s affinity to β1-integrins

**Figure 5:**
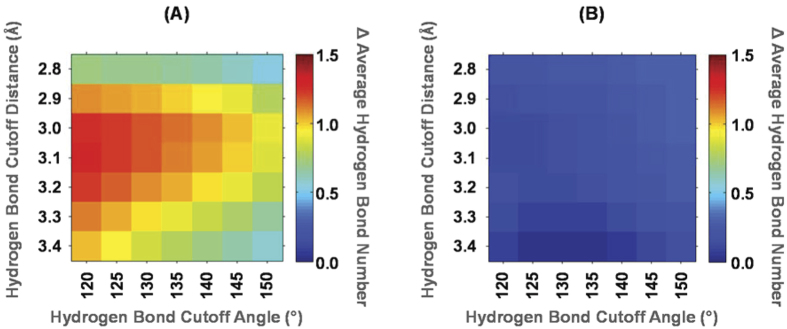
The statiscal hydrogen bond profile among talin2^WT^/integrin, talin1^WT^/integrin and talin1^C336S^/integrin complexes. (**A**) The difference of average inter-molecular hydrogen bond number between talin2^WT^/integrin and talin1^WT^/integrin. As the difference is dependent on the threshold of hydrogen bond definiation, the profile of various hydrogen bond distances and angle cutoffs is shown. Here the hydrogen bond distance is defined as the distance between hydrogen bond donor and acceptor, while the hydrogen bond angle is defined as the angle between donor, hydrogen, and acceptor. For a typical hydrogen bond distance cutoff of 3.0 Å and angle cutoff of 120°, talin2^WT^/integrin complex has about 1 more inter-molecular hydrogen bond than talin1^WT^/integrin complex. (**B)** The difference of the average inter-molecular hydrogen bond number between talin2^WT^/integrin and talin1^C336S^/integrin. It could be observed that these two complexes form an inter-molecular hydrogen bond in a similar level.

Since C336 in talin1^WT^ alters the conformation of β-sheet 1, the orientation of β1-integrin tail binding to talin1^WT^ is significantly shifted from its binding status in talin2^WT^/integrin complex. Therefore, the interaction pairs involved in the binding interface of talin1^WT^/integrin and talin2^WT^/integrin complex are much different. Besides, vibration of β1-integrin tail in these complex structures is also observed during 40 ns simulations, which implies that the static comparison of interaction pairs would be biased. We calculated the statistical hydrogen bond profile of the binding interface to reveal the difference among the three binding complexes. Figure 5 depicts is the heat map that shows the change in the hydrogen bond profile of talin1^WT^/integrin and talin1^C336S^/integrin compared with talin2^WT^/integrin. More hydrogen bonds are formed by Talin2^WT^/integrin complex than talin1^WT^/integrin complex, supporting the statement that β1-integrin binds to talin2^WT^ with higher affinity than binding to talin1^WT^ (Fig. 5A). Moreover, substitution of talin1 C336 with Ser promotes hydrogen bond formation between talin1 and β1-integrin (Fig. 5B). This is consistent with the experiment result that substitution of talin1 C336 with Ser enhances the affinity of talin1 to β1-integrin tails. Based on the MD simulation, two hydrogen bonds between β1- β2 loop of talin and β1-integrin tails, i.e., hydrogen bonds between Tln-K324/327 and Int-D759, and between Tln-N323/326 and Int-R760 (Tln refers to talin, Int for β1-integrin tails), are observed in typical snapshots of talin1^C336S^/integrin and talin2^WT^/integrin complexes (Fig. 4G,I), but are missing in talin1^WT^/integrin complex (Fig. 4H),. According to the tracked distance of this hydrogen bond depicted in Fig. 4B, the average distance (in the last 10 ns of simulation) of hydrogen bond between Tln-K324/327 and Int-D759 is 2.02 ± 0.52 Å (frequency of hydrogen bond occurrence: 95.1%, with 2.5 Å H-O distance threshold) and 2.00 ± 0.53 Å (frequency: 90.6%) in talin1^C336S^/integrin and talin2^WT^/integrin complex, respectively, while this distance increases to 5.07 ± 1.07 Å (frequency: 0.1%) in talin1^WT^/integrin complex. Similarly, the average distance (in the last 10 ns of simulation) of the hydrogen bond between Tln-N323/326 and Int-R760 is 2.39 ± 0.46 Å (frequency: 76.2%) and 2.51 ± 0.79 Å (frequency: 63.2%) in talin1^C336S^/integrin and talin2^WT^/integrin complex, respectively, while this distance increases to 7.40 ± 1.47 Å (frequency: 0.0%) in talin1^WT^/integrin complex (Fig. 4C). Although there are many other differences among the three complexes, status of these two hydrogen bonds could be considered as key indicators of talin-integrin binding state. It should be noted that both Tln-K324/327 and Tln-N323/326 are located in the β1- β2 loop (Fig. 4G), so the conformation of the β1-β2 loop is expected to influence the interaction between talin and intergrin. In conclusion, residue C336 of talin1^WT^ disturbs the conformation of β-sheet 1 as well as β1-β2 loop, thus eliminating both hydrogen bonds between Tln-K324 and Int-D759, and Tln-N323 and Int-R760, which results in an off-switch in the orientation of β1-integrin binding and a decrease in the affinity of talin1^WT^ to β1-integrin tails. On the contrary, talin1^C336S^ adapts a conformation of the β1-β2 loop seen in talin2/integrin complex, thus restoring the hydrogen bonds between Tln-K324 and Int-D759, and Tln-N323 and Int-R760, which is expected to enhance the affinity of talin1^C336S^ to β1-integrin tails.

### The critical role of the hydrogen bonds between Tln-S339 and Tln-E353, Tln-K327 and Int-D759, and Tln-N326 and Int-R760 for talin2’s high affinity to β1-integrin tails

**Figure 6:**
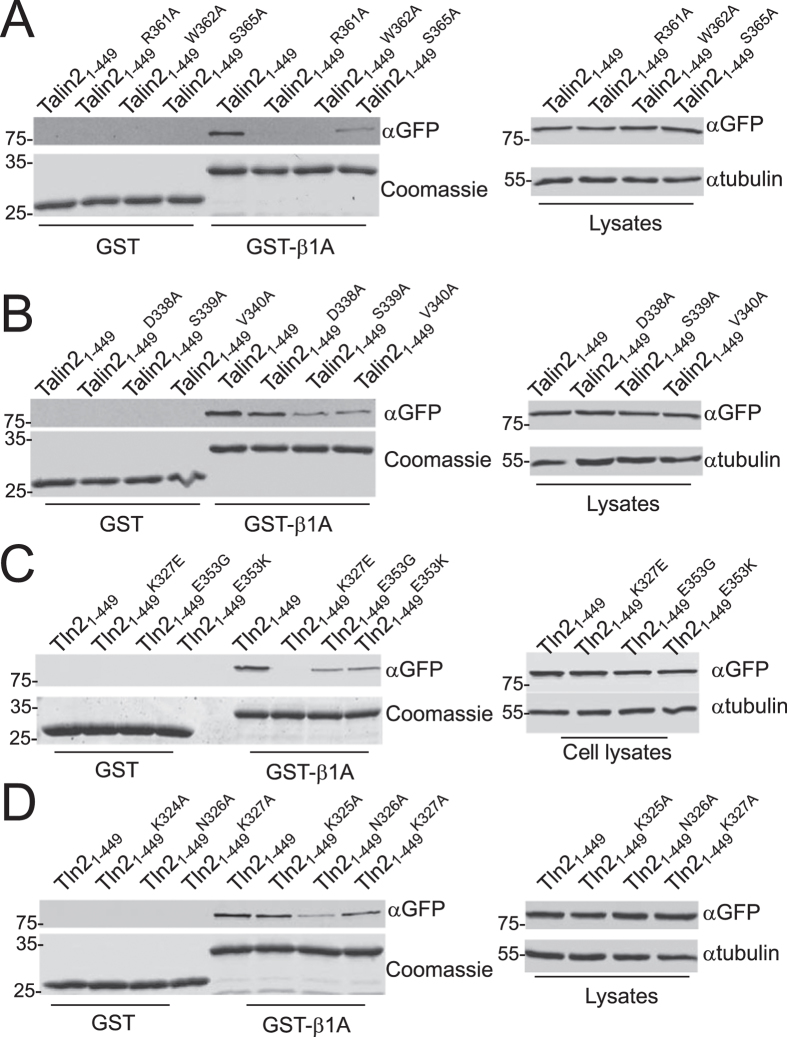
Experimental examination of the computational model. (**A**) Binding of EGFP-talin2_1–449_^WT^, -talin2_1–449_^R361A^, -talin2_1–449_^W362A^, and -talin2_1–449_^S365A^ to β1-integrin tails by GST pulldown assays. The EGFP fusion proteins were transiently expressed in CHO-K1 cells. (**B**) EGFP-talin2_1–449_^WT^, -talin2_1–449_^D338A^, -talin2_1–449_^S339A^, and -talin2_1–449_^V340A^ were transiently transfected in to CHO-K1 cells. The binding of these proteins to β1-integrin tails was determined by GST pulldown assays. (**C**) Binding of EGFP-talin2_1–449_^WT^, -talin2_1–449_^K327E^, -talin2_1–449_^E353G^, and -talin2_1–449_^E353K^ to β1-integrin tails by GST pulldown assays. (**D**) Binding of EGFP-talin2_1–449_^WT^, -talin2_1–449_^K324A^, -talin2_1–449_^N326A^, and -talin2_1–449_^K327A^ to β1-integrin tails by GST pulldown assays. Cropped blot/gel images are shown. The full-length blot/gel images are included in Supplementary Fig. S4.

Next, we set out to verify our computational model. The phosphotyrosine binding (PTB)-like domain is responsible for talin1 binding to β1-integrin tails. To prove whether this is true for talin2, R361, W362, and S365 within the talin2 PTB-like domain were substituted with Alanine, and the talin2 mutants and the WT were transiently transfected into CHO-K1 cells. The binding of these proteins to β1-integrin tails was determined using GST pulldown assays. As shown in Fig. 6A, mutation at any of these residues caused dramatic reduction in talin2 binding to β1-integrin tails, indicating that talin2 and talin1 employ the same motif to bind β1-integrin tails.

To confirm the critical role of S339 in modulating talin2 binding to β1-integrin tails, S339 and the adjacent residues D338 and V340 were mutated to Ala. These mutants and the WT were transfected to CHO-K1 cells and their binding to β1 A tails was examined. Substitution of either S339 or V340 with Ala significantly diminished talin2 binding to β1-integrin tails; Substitution of D338 with Ala also slightly decreased talin2 binding (Fig. 6B).

Based on our computational model, talin2 S339 and E353 form a hydrogen bond between the β3 and β4 strand of β-sheet 1, which promotes talin2 binding to β1-integrin tails. To verify the role of E353 in mediating the talin2 β1-integrin interaction, talin2 E353 was substituted with Gly and Lys, respectively, and the interaction of these mutants with β1-integrin tails was determined. Substitution of talin2 E353 with either Gly or Lys significantly diminished the binding of talin2 to β1-integrin tails (Fig. 6C), suggesting that the hydrogen bond between S339 and E353 is required for the high affinity of talin2 to β1-integrin tails.

To know whether the hydrogen bond between talin2 K327 and β1-integrin D759 is critical for the talin2-integrin interaction, talin2 K327 was mutated to Glu and Ala, respectively, and the binding of these mutants to β1-integrin tails was examined. Substitution of K327 with Glu abolished the interaction of talin2 with β1-integrin tails (Fig. 6C), and substitution of K327 with Ala also reduced the binding of talin2 (Fig. 6D). In addition, substitution of N326 with Ala significantly diminished the interaction of talin2 with β1-integrin tails (Fig. 6D), which also supports the predicted hydrogen bond between N326 of talin2 and R760 of β1-integrin. Taken together, the hydrogen bonds between talin2 S339 and E353 and between talin2 K327 and β1-integrin D759 are critical for talin2 to maintain its high affinity to β1-integrins.

**Figure 7:**
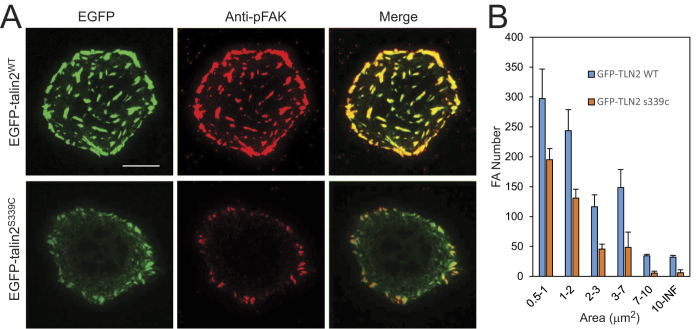
Substitution of talin2 S339 with Cys diminished focal adhesion formation. Talin2-null U2 OS cells were transfected with EGFP-talin2^WT^ and -talin2^S339C^, respectively, plated on fibronectin (5 μg/ml), fixed with paraformaldehyde, and stained with anti-phospho-FAK[pY397] antibody. Focal adhesions were examined with a TIRF microscopy. Scale bar, 20 μm. (**A**) The distribution of talin2, talin2^S339C^, and phospho-FAK in U2 OS cells. (**B**) Area distribution of phospho-FAK staining in U2 OS cells. Data are mean ± SEM of 3 experiments. In each group, FAs from 20 cells were analyzed and plotted.

Talin2 is a focal adhesion protein. To know whether talin2 S339 is critical for focal adhesion formation, talin2-null human osteosarcoma cells U2 OS were transfected with EGFP-talin2^WT^ and –talin2^S339C^, respectively. Cells were plated on glass-bottom dishes that were pre-coated with fibronectin (5 μg/ml), fixed, and stained with an anti-phospho-FAK antibody. The images of EGFP and phospho-FAK were recorded with a TIRF microscope. EGFP-talin2^WT^ was co-localized with phospho-FAK, whereas EGFP-talin2^S339C^ was deficient in focal adhesion formation and had a diminished co-localization with phospho-FAK (Fig. 7A,B), suggesting that a strong interaction of talin2 with β1-integrins is essential for efficient focal adhesion assembly.

## Discussion

We found that talin1 and talin2 interacted with β1A-integrin tails with K_d_ of 0.88 and 0.35 μM, respectively. Substitution of S339 with Leucine completely disrupted talin2 binding to β1-integrins, and mutation of S339 to Cys also diminished its binding, indicating that S339 is critical for talin2 binding to β1-integrins. Our findings suggest that the incapacity of S339L mutant binding to β-integrins could be the pathological cause of fifth finger camptodactyly.

Substitution of talin1 C336 with Ser caused only a small increase in talin1’s affinity (Fig. 1), but C336S mutant bound to β integrin tails more strongly than talin1^WT^ when talin1 and C336S were expressed in mammalian cells^33^. Similar result was observed as comparing the binding of talin2^WT^ and talin2^S339C^. These results suggest that talin and mutants from mammalian cells may have post-translational modifications that influence their binding to β–integrin tails. However, the possibility that proteins from cell lysates have superior folding compared to those from bacteria cannot be ruled out.

Previous study reported that the F3 domains of talin1 and talin2 bound to β1 A tails with K_d_ of 491 and 652 μM, respectively, as measured by NMR^11^. Our findings are different from the results in this report. As we discussed previously, the difference could be caused by using different fragments of the talin head domains and different binding buffers and blocking reagents in the assays^33^. The folding of talin head domains purified from bacteria could also cause this difference. Although our gel filtration chromatography analysis indicates that these proteins are folded (Supplementary Fig. S1), this method does not prove that the proteins are folded and post-translationally modified exactly as in their natural host. Our unpublished data show that talin head domains induced by IPTG at 37 °C had higher β1 A integrin binding capacities than those induced at 19 °C, probably because the expression of chaperones that control protein folding are inhibited at a low temperature^37^,^38^. We used talin head domains that were induced by IPTG at 37 °C in our affinity assays. We do not have evidence that the proteins studied here would have been folded better than in other studies. However, our affinity assays with purified recombinant proteins from E. coli are consistent with the pulldown assay results conducted using mammalian cell lysates. Talin head domain purified from CHO cells will be useful to further clarify this difference.

Our computational modeling results show that talin2 S339 forms hydrogen bonds with E353, which is critical for key hydrogen bonds between talin2 K327 and β1-integrin D759, and between talin2 N326 and β1-integrin R760 (Fig. 4D,G). These hydrogen bonds were not observed in the talin1/integrin complex (Fig. 4E,H). Mutation at any of these residues significantly diminished the binding of talin2 with β1-integrin tails (Fig. 6). However, substitution of talin1 C336 with Ser promoted the formation of these hydrogen bonds (Fig. 4F,I), accompanying an increase in its affinity toward β1-integrin tails. Interestingly, mutation of talin2 S339 to Leu caused a significant reduction in its binding to β1-integrin tails (unpublished data) and Fifth Finger Camptodactyly, suggesting that the reduction in the talin2-integrin interaction could be the cause for the genetic disease. These results suggest that the hydrogen bonds between talin2 S339 and E353 play a critical role in maintaining talin2’s high affinity to β1-integrin. Because these residues are located outside of the well-characterized β1-integrin-binding motif, it is possible to design a small molecule to diminish talin2 binding to β1-integrins without affecting talin1.

Besides mediating β-integrin interaction, talin2 K327 may also be involved in other functions of talin2. Previous studies show talin1 K324, which is aligned with K327 on talin2, to be a key residue for talin1 binding to PIP2 and is essential for integrin clustering^39^. This residue also mediates the head-and-rod auto-inhibition of talin1^40^,^41^,^42^. Studies have also shown that PIP2 binds to talin1 and disrupts the head-and-rod interaction, thus causing talin1 activation^40^,^43^. It is likely that talin2 K327 is a key residue for the talin2-PIP2 interaction and is also involved in the head-and-rod auto-inhibition of talin2. However, it is unknown whether binding to PIP2 disrupts or enhances talin2-β1-integrin interaction.

Talin2 N236 is another key residue that mediates talin2-β1-integrin interaction. In fact, substitution of talin2 N326 with Ala had more significant effect on the binding of talin2 to β1-integrin tails than that of K327 with Ala (Fig. 6D). Although substitution of talin2 K327 with Glu disrupted the interaction of talin2 with β1-integrin tails (Fig. 6C), this could be caused by the perturbation of the negative charge of the Lys residue on talin2 conformation. Since talin2 K327 may involve in PIP2 interaction, the hydrogen bond between Tln-N326 and Int-R760 could play a key role in the talin2-β1 integrin interaction, especially in the presence of PIP2.

## Materials and Methods

### Reagents

Anti-FAK[pY397] was from BD Biosciences. Anti-tubulin antibody was from Sigma. DyLight 549 conjugated goat anti-mouse IgG (H + L) was from Thermo Scientific. Fibronectin and recombinant human EGF were from Akron Biotech; Growth factor reduced Matrigel was from BD Bioscience. Pfu Ultra was from Agilent Technologies. Cold Fusion Cloning Kit was from System Biosciences (Palo Alto, CA). Anti-GFP monoclonal antibody and Safectine RU50 transfection kit were purchased from Syd Labs (Malden, MA). DNA primers were synthesized by Sigma-Aldria.

### Plasmid construction

The full-length pEGFP-talin2 WT was subcloned by the following steps: 1) DNA fragments encoding residues 1–1159 of human talin2 were amplified by Pfu Ultra-based PCR using human talin2 cDNA clone as template and 5′-atg cac tcg agc tat ggt ggc cct gtc ctt aaa gat ttgt-3′/5′-act gag gta ccg tct cga gca gaa tct aac atg gca t-3′ as primers and subcloned into pEGFP-C1 via Xho1/Kpn1 sites; 2) fragments encoding residues 1160–2543 of talin2 were amplified using human cDNA from U2 OS cells and 5′-ggc tgc atc gac aac cga ccc c-3′/5′-tat tat cta gat tag ccc tca tct tcc ctc agc tc-3′ and subcloned into the resulted plasmid in step 1 via Not1/Xba1 sites. pEGFP-talin2_1–449_ was generated by amplifying DNA fragments encoding residues 1–449 using 5′-atg cac tcg agc tat ggt ggc cct gtc ctt aaa gat ttg t-3′/5′-ggg ccc gtc gac tat gag ccg tgc tct gcc ttc cc-3′ as primers and subcloning into pEGFP-C1 vector via Xho1/Sal1 sites. pEGFP-talin2_1–449_^S339C^ was created by pfu Ultra-based PCR using pEGFP-talin2_1–449_ as template and 5′-gga tca cca aag act gtg tga tgc gcg tgg-3′/5′-cca cgc gca tca cac agt ctt tgg tga tcc-3′ as primers. pEGFP-talin2_1–44_^9R361A,^ -talin2_1–449_^W362A^, and -talin2_1–449_^S365A^ was generated by pfu Ultra-based PCR using pEGFP-talin2_1–449_ as template and 5′-ctc acc accgtc aag gcc tgg gca gcc tca ccc-3′/5′-ggg tga ggc tgc cca ggc ctt gac ggt ggt gag-3′, 5′-acc acc gtc aag cgc gcg gca gcc tca ccc aag-3′/5′-ctt ggg tga ggc tgc cgc gcg ctt gac ggt ggt-3′, and 5′-aag cgc tgg gca gcc gca ccc aag agc ttc aca-3′/5′-tgt gaa gct ctt ggg tgc ggc tgc cca gcg ctt-3′ as primers, respectively. pEGFP-talin2_1–449_^D338A^, -talin2_1–449_^S339A^, and -talin2_1–449_^V340A^ was created by PCR using 5′-ctg ggg atcacc aaa gcc tcg gtg atg cgc gtg-3′/5′-cac gcg cat cac cga ggc ttt ggt gat ccc cag −3′, 5′-ggg atc acc aaa gac gcg gtg atg cgc gtg gat-3′/5′-atc cac gcg cat cac cgc gtc ttt ggt gat ccc-3′, and 5′-atc acc aaa gac tcg ggg atg cgc gtg gat gag −3′/5′-ctc atc cac gcg cat ccc cga gtc ttt ggt gat-3′ as primers, respectively. pEGFP-talin2_1–449_^K325A^, -talin2_1–449_^N326A^, and -talin2_1–449_^K327A^ was created by PCR using 5′-aag gag aag atg aaa ggc gcg aac aag ctg gtg cct cg-3′/5′-cga ggc acc agc ttg ttc gcg cct ttc atc ttc tcc tt-3′, 5′-aga aga tga aag gca agg cca agc tgg tgc ctc gcc-3′/5′-ggc gag gca cca gct tgg cct tgc ctt tca tct tct-3′, and 5′-gat gaa agg caa gaa cgc gct ggt gcc tcg cct g-3′/5′-cag gcg agg cac cag cgc gtt ctt gcc ttt cat c-3′ as primers, respectively. pEGFP-talin2_1–449_^E353G^, -talin2_1–449_^E353K^, and -talin2_1–449_^K327E^ was created by PCR using 5′-agg aag tgc tgc agg ggt ggc ccc tca cca c-3′/5′-gtg gtg agg ggc cac ccc tgc agc act tcc t −3′, 5′-agg aag tgc tgc aga agt ggc ccc tca cca c −3′/5′-gtg gtg agg ggc cac ttc tgc agc act tcc t −3′, and 5′-atg aaa ggc aag aac gag ctg gtg cct cgc c −3′/5′-ggc gag gca cca gct cgt tct tgc ctt tca t −3′ as primers, respectively. The full-length pEGFP-talin2^S339C^ was created by digesting full-length pEGFP-talin2 with BsrG1/EcoRV and ligating the resulting larger fragment with the smaller fragments from pEGFP-talin2_1–449_^S339C^. pQE-talin1_1–446_ was generated by amplifying the DNA fragments using human talin1 cDNA as template and 5′-ggg ccc gag ctc atg gtt gca ctt tca ctg aag atc ag-3′/5′-ggg ccc gtc gac tta aga gcc atg ctc cac ttt ccc c-3′ as primers and subcloning into pQE-30 vector via Sac1/Sal1. pQE-talin1_1–446_^C336S^ was created by PCR using pQE-talin1_1–446_ as template and 5′-cat cac caa gga gag tgt gat gcg ag-3′/5′-ctc gca tca cac tct cct tgg tga tg −3′ as primers. pQE-talin2_1–449_ and -talin2_1–449_^S339C^ were generated by amplifying the DNA fragments using 5′-atg cag aat cca tgg tgg ccc tgt cct taa aga ttt gt-3′/5′-ggg ccc gtc gac tat gag ccg tgc tct gcc ttc cc-3′ as primers, pEGFP-talin2_1–449_ and pEGFP-talin2_1–449_^S339C^ as templates, and subcloning into pQE-30 vector via Sac1/Sal1 and BamH1/Sal1, respectively. All plasmids were sequenced by Eurofins MWG Operon (Huntsville, AL).

### Protein interaction assays

CHO-K1 cells were transfected with pEGFP-talin1_1–433_, -talin1_1–446_, -talin1_1–449_, or their mutants. At 28 h post-transfection, the cells were harvested in lysis buffer A (50 mM Tris-HCl pH 7.4, 1% NP-40, 150 mM NaCl, 1 mM EDTA and a protease inhibitor cocktail). Cell lysates were cleared by centrifugation and incubated with glutathione–Sepharose beads loaded with GST or GST-β1-integrin tails at 4 °C for 2 h. The beads were washed with the lysis buffer four times and resuspended in SDS-sample buffer. Samples were analyzed using SDS–PAGE and transferred to nitrocellulose membrane for the detection of interacting proteins.

The binding of purified His-tagged proteins to GST-β1-integrin tails was performed in lysis Buffer A containing 0.5 mg/ml bovine gelatin unless where specified. Bound proteins were separated using SDS-PAGE, stained with Coomassie blue. The gels were scanned with LI-COR Infrared Imager using 700 nm channel. Protein bands was quantified by analyzing inverted images using ImageJ as described previously^20^,^44^, and calibrated with standards run on the same gels. The binding curves and the K_d_ were analyzed with SigmaPlot.

### Molecular Dynamic Simulations

For the three talin/integrin complex systems, i.e., talin2^WT^/integrin, talin1^WT^/integrin, and talin1^C336S^/integrin, Amber 12 package (Case, D.A. *et al*., University of California, San Francisco, 2012) is used to perform energy minimization and MD simulations in TIP3P solvent environment in a way similar to that used in our previous studies^35^,^36^,^45^.

### Talin2/integrin complex structural model

X-ray crystal structure of *Mus musculus* talin2 and *Homo sapiens* β1D-integrin complex (PDB entry as 3G9W with resolution of 2.16 Å) reveals the interaction between F2-F3 domains (residue #200–406) of talin2 and tail region (residue #750–789) of β1D-integrin^11^. Sequence identity between *Mus musculus* talin2 (accession number as Q71LX4 in protein sequence database of UniProt^46^) and *Homo sapiens* talin2 (accession number as Q9Y4G6) is 92.0%. However, sequence of F2-F3 domains of *Mus musculus* talin2 is fully conserved with corresponding domains of *Homo sapiens* talin2. Thus, the reported *Mus musculus* talin2 and *Homo sapiens* β1D-integrin complex structure could be considered equivalent to *Homo sapiens* complex structure. For convenience, the talin/integrin complex model in this manuscript refers to the truncated complex structure, i.e., F2-F3 domains of talin complex with tail region of β1D-integrin, unless otherwise noted.

### Building the talin1/integrin complex structural model

Talin1/integrin model is constructed based on talin2/integrin structure with homology modelling method implemented by MODELLER module of Discovery Studio 2.5 (Accelrys Inc., San Diego, CA, 2009). According to the sequence alignment performed by using PROMALS3D server (http://prodata.swmed.edu/promals3d/)^47^, sequence identity between wild type *Homo sapiens* talin2 (talin2^WT^) and *Homo sapiens* talin1 (talin1^WT^, accession number as Q9Y490) is 76.0%. Furthermore, the aligned sequences (Fig. 3A) indicate that the F2-F3 domains of talin2 are homologous to the corresponding F2-F3 domains of talin1 with a much higher sequence identity of 86.7%. Based on the information from the sequence alignment, the initial coordinates of atoms in the conserved regions of talin1 and whole tail region of β1-integrin are directly transformed from the template structure of talin2/integrin complex, whereas the initial coordinates for the non-conserved residues of talin1 are mutated from these of the corresponding residues in the template. The model with best DOPE scores^48^ is selected as the initial talin1/integrin complex structure (see below). Moreover, the *in silico* C336S mutant of talin1 (talin1^C336S^) and wild type β1-integrin complex is obtained by using the mutagenesis function of PYMOL (http://www.pymol.org/)^49^ based on the constructed talin1^WT^/integrin complex structure.

### Cell culture and transfection

CHO-K1 Chinese hamster ovary cells and U2 OS human bone osteosarcoma cells were from the American Type Culture Collection and were maintained in DMEM medium (Corning Inc.) containing 10% fetal bovine serum (FBS), penicillin (100 U/ml) and streptomycin (100 μg/ml). CHO-K1 and U2 OS cells were transfected with Safectine RU50 according to the manufacturer’s protocol.

### FA staining

Endogenous talin2 in U2 OS cells was ablated using CRISPR techniques and EGFP-talin2^WT^ and –talin2^S339C^ were then stably re-expressed in talin2-null cells, respectively, as described previously^33^. The resulted cells were plated on glass-bottom dishes that were pre-coated with fibronectin (5 μg/ml) and cultured for 4 h. The cells were fixed with 4% paraformaldehyde for 15 min, permeabilized for 15 min with 0.5% Triton X-100, and then blocked with 5% BSA in PBS for 1 h. The cells were then incubated with anti-phospho-FAK[pY397] antibody, washed with PBS, and then incubated with Dylight550-labeled goat anti-mouse secondary antibody. After washing with PBS, the images of EGFP and phospho-FAK[pY397] were acquired with a Nikon Eclipse Ti TIRF microscope equipped with a 60 × , 1.45 NA objective, CoolSNAP HQ2 CCD camera (Roper Scientific). Focal adhesion area distribution was analyzed with Nis-Elements.

## Additional Information

**How to cite this article:** Yuan, Y. *et al*. The molecular basis of talin2’s high affinity toward β1-integrin. *Sci. Rep.* **7**, 41989; doi: 10.1038/srep41989 (2017).

**Publisher's note:** Springer Nature remains neutral with regard to jurisdictional claims in published maps and institutional affiliations.

## Supplementary Material

Supplementary Information
