# Supplementary material for: The molecular basis of talin2’s high affinity toward β1-integrin
Source: Sci Rep. 2017 Feb 3;7:41989. doi: 10.1038/srep41989 (PMC5290461; doi:10.1038/srep41989)
Supplement: Supplementary Information [file srep41989-s1.pdf]

Supplementary information

The molecular basis of talin2's high affinity toward β1-integrin

Yaxia Yuan, Liqing Li, Yanyan Zhu, Lei Qi, Latifeh Azizi, Vesa P. Hytönen, Chang-Guo Zhan, and Cai Huang

Method for determining molecular weights by gel filtration chromatography

Molecular weight determination was performed using a Malvern Zetasizer µV instrument (Malvern Instruments Ltd, Worcestershire, UK) running a Static Light Scattering (SLS) and Dynamic Light Scattering (DLS) methods. Protein were analyzed using a liquid chromatography instrument (CBM-20A, Shimadzu Corporation, Kyoto, Japan) equipped with autosampler (SIL-20A), UV-VIS (SPD-20A) and fluorescence detector (RF-20Axs). Data were processed using Lab Solution Version 5.51 (Shimadzu Corporation) and OmniSec 4.7 (Malvern Instrument Ltd) softwares. Samples (50µg) were injected on a Superdex 200 10/300 GL column (GE Healthcare, Uppsala, Sweden). The column was equilibrated with 50mM NaH_3PO_4, 150mM NaCl pH 7.2 running buffer. Runs were performed with flow rate of 0.5 ml/min at 20 °C. Molecular weights were calculated by calibrating the system using BSA for the calculating of light-scattering intensity of the eluting protein.

Supplementary Table1. Molecular weight (SLS) obtained from HPLC analysis from four parallel runs

Proteins	Elution volume (ml) (SEC-LS)	MW (Da) by SLS	MW (Da) theoretical
His tagged-Talin1$_{1\text{-}446}^{WT}$	13.285	59,058	53315.2
His tagged-Talin1$_{1\text{-}446}^{C336S}$	13.172	58,532	53299.1
His tagged-Talin2$_{1\text{-}449}^{WT}$	13.292	58.839	53325.8
His tagged-Talin2$_{1\text{-}449}^{S339C}$	13.636	59.411	53341.8

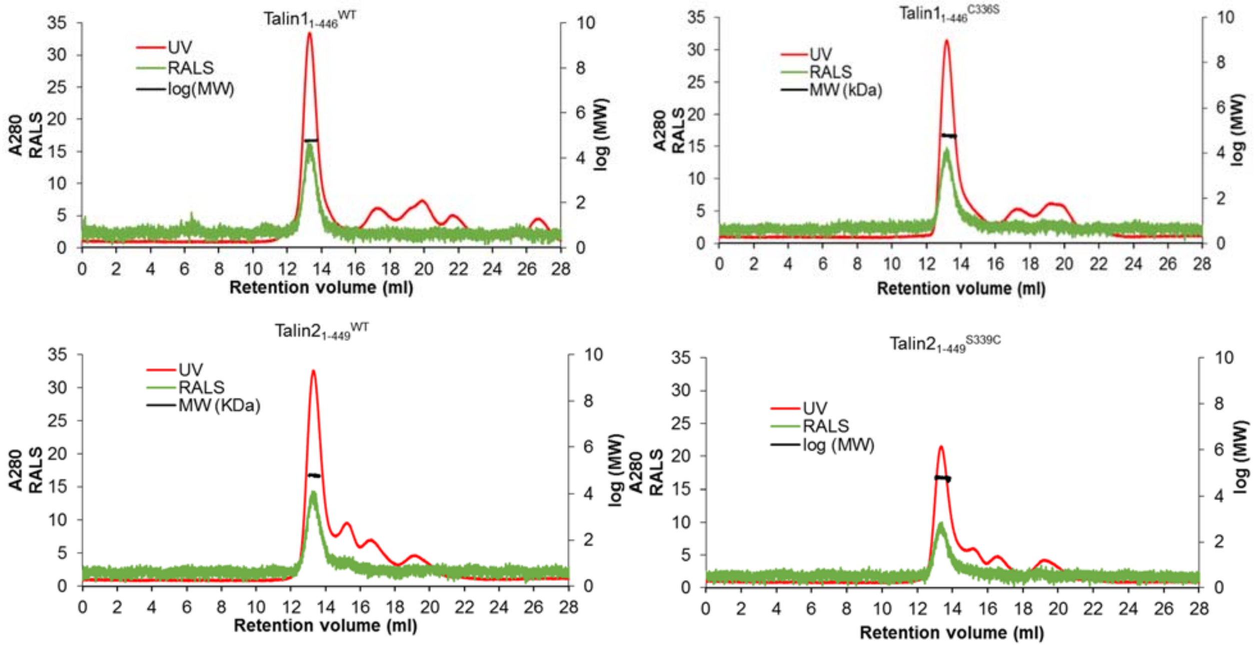

Supplementary Fig. S1. Measurement of the molecular weights of talin1$_{1\text{-}446}^{WT}$, talin1$_{1\text{-}446}^{C336S}$, talin2$_{1\text{-}449}^{WT}$, and talin2$_{1\text{-}449}^{S339C}$ by gel filtration assays. Purified His-tagged proteins were run using Superdex 200 10/300 GL column with flow rate 0.5 ml/min in 50mM NaH3PO4, 150mM NaCl pH 7,2 buffer. The left Y-axis shows the UV (280nm) and LS signal intensities. Molecular weight (MW) calculated from LS signal are shown on the right Y-axis. BSA was used for the LS detector calibration.

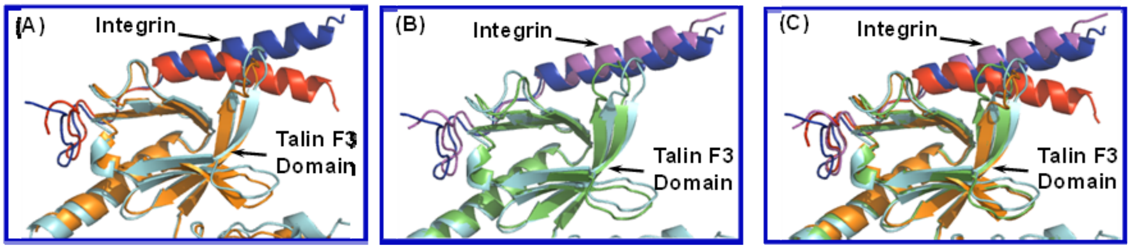

Supplementary Fig. S2. Average structure of Talin/Integrin complex. (A) Superimposition of the average structure of Talin2-WT/Integrin and Talin1-WT/Integrin complex. The average structure is derived from 40 ns MD trajectory. For Talin2-WT/Integrin complex, Talin2-WT and Integrin are colored in cyan and blue, respectively. For Talin1-WT/Integrin complex, Talin1-WT and Integrin are colored in golden and red, respectively. Talin2 and Integrin are represented as cyan and blue ribbons, respectively. (B) Superimposition of the average structure of Talin2-WT/Integrin and Talin1-C336S/Integrin complex. For Talin1-C336S/Integrin complex, Talin1-C336S and Integrin are colored in green and purple, respectively. (C) Superimposition of average structure of all three Talin/Integrin complex.

Fig 1B

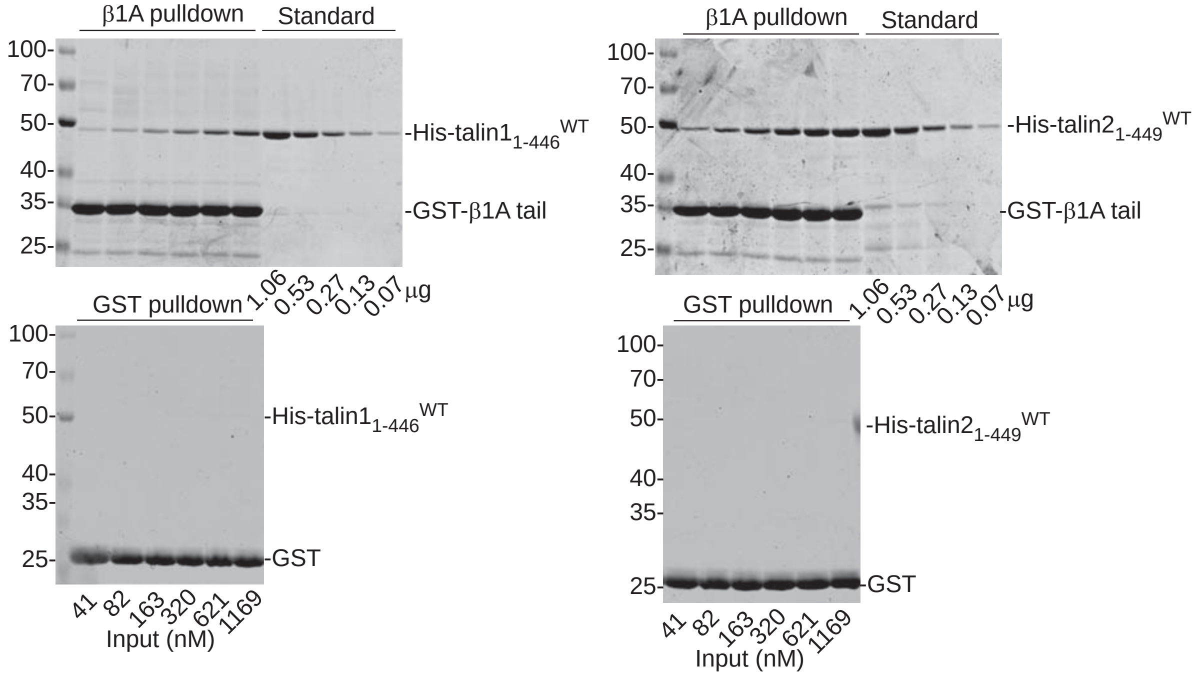

Fig 1E

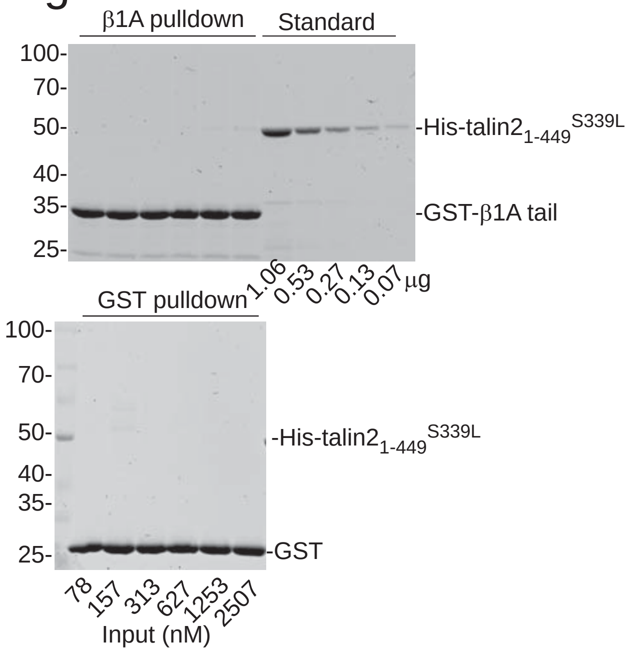

Supplementary Fig. S3. The full-length gels of Fig. 1

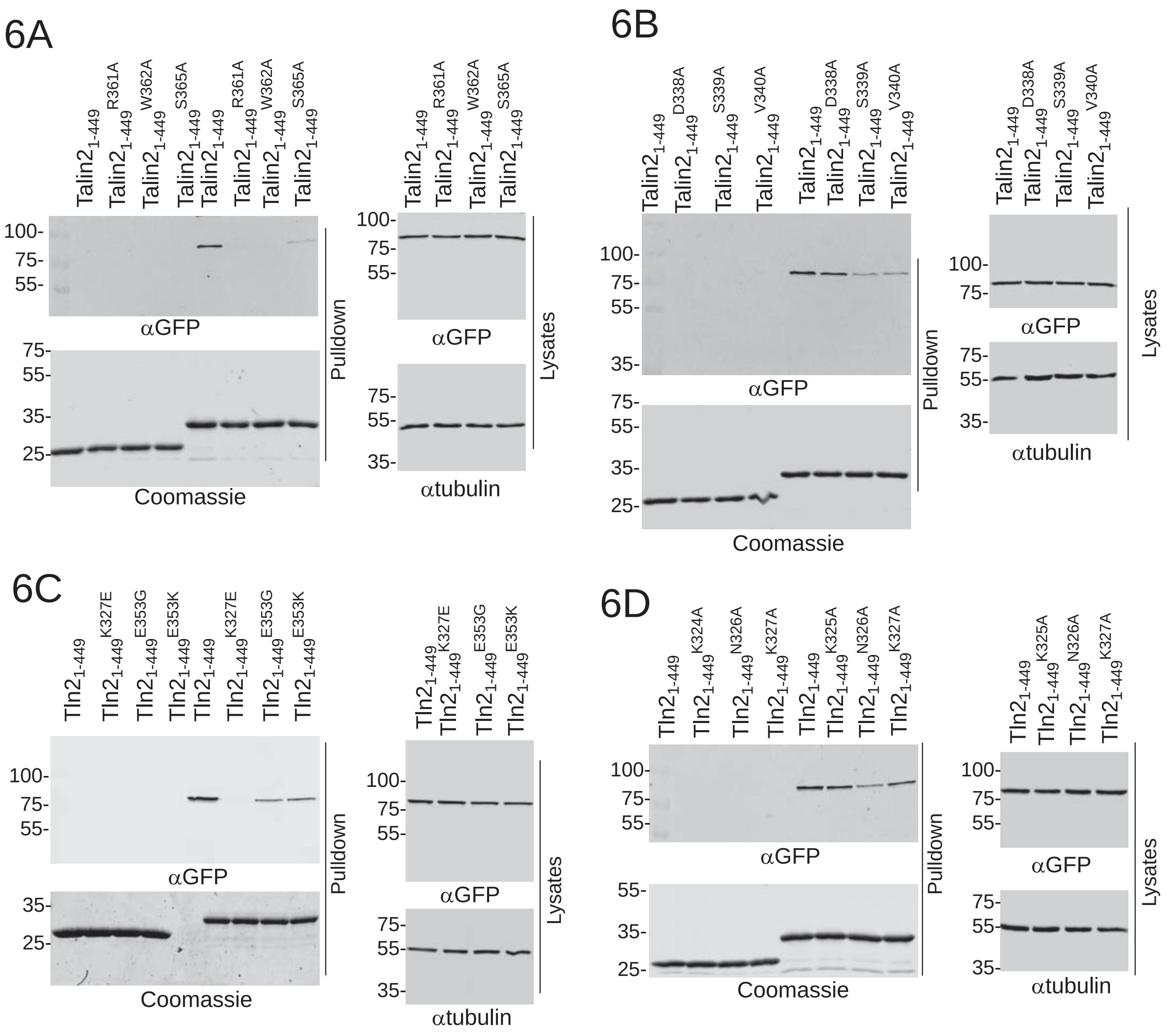

Supplementary Fig. S4. The full-length gels and blots of Fig. 6
